# Aphorisms

**Published:** 1899-02

**Authors:** 


					﻿OPEN-AIR TREATMENT OF PHTHISIS AS PRACTICED
IN GERMAN SANITARIA.
Impure or “rebreathed” air is the principal predisposing
cause of phthisis; the leading principle of the treatment,
described by Dr C. T. Williams (British Medical Journal and
American Medico-Surgical Bulletin), is that the consumptive
should pass the. greater part of his time in the open air, pro-
tected from weather, and as a rule in the prone position, and
that at night he should sleep with windows open. This treat-
ment is carried on either in covered balconies or terraces with
one side open or in pavilions, some of which are arranged to
rotate, the invalid being thus enabled to be always protected
from wind and exposed to sunshine. The covered terraces are
of considerable depth and height; the drifting rain and snow
are kept out by curtains, and the too intense sunheat by
blinds. The patient lies out from seven to eleven hours a day,
only moving for meals and exercise, and going indoors at
night. Thus protected, all kinds of weather are well borne,
provided only the patients are well covered up in furs and
wraps.
To resist cold the prone position is far better than sitting,
probably because of the equalizing effect it has on the circu-
lation of the body and the less strain it exerts on the heart.
The author has found patients lying in comfort in the open
nir at a temperature of 4° F. ; on feeling their hands and feet
he found them perfectly warm. Thus the patient is hardened
against fresh cold, his appetite is increased, his sleep is pro-
moted, his night sweats reduced, and his pyrexia is lowered.
There are two objections to the prone position ; first, it is not
well adapted for clearing cavities by expectoration, some of
the sputum being liable to return to another bronchus, thus
making a new focus of infection; second, in incipient disease
it is impossible to maintain the muscles and the functions in
proper order without exercise. Therefore, some physicians
use this Liegenhalle system alone, while others add to it liill-
•climbing and other forms of pulmonary gymnastic®. The ex-
ercises are regulated, the patient being enjoined to ascend
slowly, not to talk while climbing, to breathe through the
nose, and to stop short of actual fatigue. During steady
walking five or six deep breaths are to be taken every 100 or
150 paces, breathing through the nose, or when lying in the
■open air taking from ten to twelve deep breaths every five or
ten minutes.
The patient is stuffed with a rich and varied diet, and hy-
dropathy is used, especially douches to the thorax to stimulate
respiration. The floors of these sanitaria are covered with
linoleum, the walls are painted with oil-colors, or are inlaid
with wooden panels or papered with washable material ; there
is no sweeping done, but swabbing, instead, with damp cloths.
The sputum is spat into vessels and afterward destroyed; it
is frequently examined and when no bacilli can be found 1 cc.
of the sputum is injected under the skin of a guinea-pig ; and
if in from three to six weeks the animal has not contracted
tuberculosis the patient is considered fit to return home.—
Practical Medicine.
APHORISMS.
Affirmation and argument are alike in so far as*they begin
with the same letter.
A man would be thought insane if he talked about the
danger of a cold catching a baby when its teeth are cutting
it. But how does it stand as a matter of fact?
Punctuality is the thief of time.
The latest dictum of English psycholögy seems to be that a
“fellah don’t know what he does know if he only knew it,,
don’t cher know.”
The man who has “private opinions” will stand watching.
Why suffer from excessive fat when obesity and even hyper-
lipomatosis cost just the same?
“To the pure all things are pure,” doesn’t necessarily apply
to the city analyst.
Better do it because it is needed than because it is Friday.
The smallpox patient is not so much to be pitied to-day as
fifty years ago.
Don’t be well-meaning.
Figures don’t lie, but for the matter of that umbrellas don’t
borrow.
Science has but little room for superlatives.
Oid, pseudo, and the potential mood are great aids in diag-
nosis.
Why don’t the good people who pray for rain include hot
winters and icy summers?—Cleveland Medical Journal.
				

